# Spiral magnetic order and pressure-induced superconductivity in transition metal compounds

**DOI:** 10.1038/ncomms13037

**Published:** 2016-10-06

**Authors:** Yishu Wang, Yejun Feng, J.-G. Cheng, W. Wu, J. L. Luo, T. F. Rosenbaum

**Affiliations:** 1 Division of Physics, Mathematics, and Astronomy, California Institute of Technology, Pasadena, California 91125, USA; 2 The Advanced Photon Source, Argonne National Laboratory, Argonne, Illinois 60439, USA; 3 Beijing National Laboratory for Condensed Matter Physics and Institute of Physics, Chinese Academy of Sciences, Beijing 100190, China; 4 Collaborative Innovation Center of Quantum Matter, Beijing 100190, China

## Abstract

Magnetic and superconducting ground states can compete, cooperate and coexist. MnP provides a compelling and potentially generalizable example of a material where superconductivity and magnetism may be intertwined. Using a synchrotron-based non-resonant X-ray magnetic diffraction technique, we reveal a spiral spin order in MnP and trace its pressure evolution towards superconducting order via measurements in a diamond anvil cell. Judging from the magnetostriction, ordered moments vanish at the quantum phase transition as pressure increases the electron kinetic energy. Spins remain local in the disordered phase, and the promotion of superconductivity is likely to emerge from an enhanced coupling to residual spiral spin fluctuations and their concomitant suppression of phonon-mediated superconductivity. As the pitch of the spiral order varies across the 3*d* transition metal compounds in the MnP family, the magnetic ground state switches between antiferromagnet and ferromagnet, providing an additional tuning parameter in probing spin-fluctuation-induced superconductivity.

## Abstract (web summary)

The relationship between magnetic order and superconductivity is one of the central issues in unconventional superconductors. Here, Wang *et al*. report a spiral spin order in MnP and trace its pressure evolution towards superconducting order, suggesting variable spiral pitch as a mechanism to tune spin-fluctuation-induced superconductivity.

## Graphical abstract

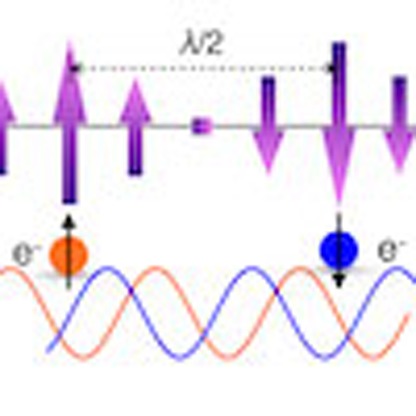

From the lodestone-based compass to modern theories of phase transitions^1^, magnetic materials have played an outsized role in revealing the shape of the world around us. The similarly venerable field of superconductivity serves as a prime example of emergent, collective behaviour in nature, with raised hopes of technological import with the discovery of exotic superconducting order in the cuprates. Magnetism and superconductivity often compete for preeminence as a material's ground state, but in the right circumstances the fluctuating remains of magnetic order can induce superconducting pairing. The intertwining of the two on the microscopic level, independent of lattice excitations, is especially pronounced in heavy fermion compounds, rare earth cuprates and iron pnictides.

Here we point out that for a helical arrangement of localized spins, a variable spiral period could provide a unique tuning process from ferromagnetic to antiferromagnetic ground state in the long and short wavelength limits, respectively. Such chemical or pressure adjustable helical order naturally provides the possibility for continuous tuning between ferromagnetically and antiferromagnetically mediated superconductivity. At the same time, phonon-mediated superconductivity is suppressed because of the local ferromagnetic spin configuration^2^ in the low-frequency spiral fluctuation modes.

The recent discovery of a superconducting phase in the transition metal compound MnP (ref. 3) opens the possibility of investigating this scenario. MnP possesses a complex pressure–temperature (*P–T*) phase diagram^3^. At ambient pressure, there is helical spin order below the Néel temperature, *T*_N_=50 K, with a wave vector **Q**=(0.117, 0, 0) (ref. 4). Under pressure, the helical order is quickly replaced by ferromagnetism at ∼1 GPa, and another magnetic state, assumed to be antiferromagnetic^3^, emerges for *P*>2 GPa. Superconductivity appears after the high-pressure magnetic phase is suppressed at *P*∼7 GPa (ref. 3). The spin structure in the high-pressure magnetic phase remains unsettled, and is under active exploration by both X-ray and neutron^5^ magnetic diffraction techniques.

We employ synchrotron-based magnetic X-ray diffraction (see ‘Methods' section) to investigate the high-pressure magnetic phases in MnP (ref. 3). This sensitive probe, suitable for 0.0002, mm^3^ single crystal volumes and diamond anvil cell techniques, directly reveals a reduced moment, incommensurate spin state at high pressure proximate to the superconducting state. This new magnetic order is most likely a magnetic helix with a tightened pitch in comparison to that at ambient pressure where superconductivity is absent. The extant data correlating magnetic pitch length and superconductivity is sparse but suggestive in the (V/Cr/Mn/Fe/Co/Ni)(P/As/Sb) family^3^,^4^,^6^,^7^,^8^,^9^,^10^,^11^,^12^,^13^,^14^,^15^,^16^,^17^,^18^,^19^ and, as discussed in detail below, we suggest this family of spiral magnets as a new venue for tunable, spin-fluctuation-mediated superconductivity.

## Results

### High-pressure spiral antiferromagnetic order

**Figure 1:**
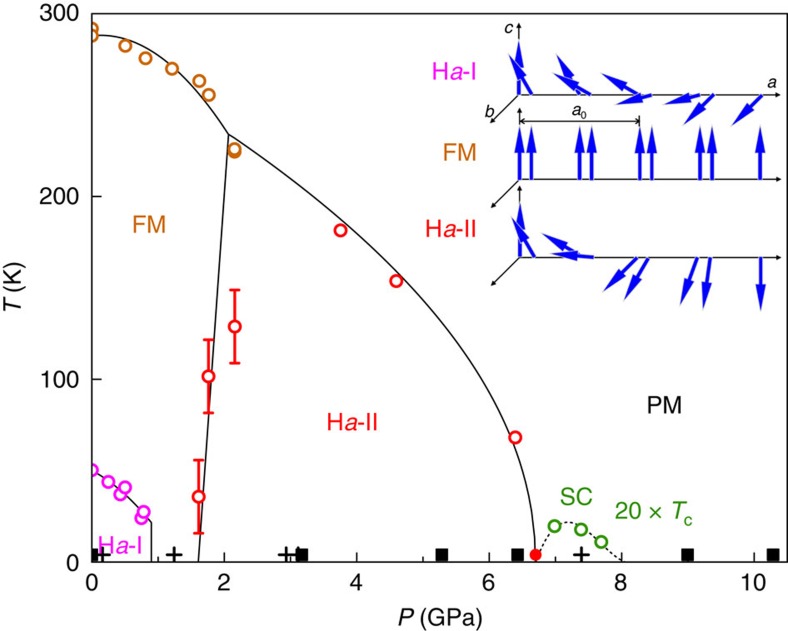
Magnetic phases of MnP. The *P–T* phase diagram includes ferromagnetism (FM), a double-helical order (H*a*-I) at low pressure^4^, a new helical order (H*a*-II) discovered at high pressure in the current work, superconductivity (SC) and paramagnetism (PM). Phase boundary data is adapted from ref. 3 (open circles) with a reduction of pressure scale by a factor of 1.12 to match our X-ray measured H*a*-II phase boundary at 4 K (filled circle). Also marked are (*P*, *T*) positions where the helical order was observed or proved null through magnetic scattering (filled squares) and where the lattice parameters are measured (crosses). The presence of multiple ferromagnetic phases^44^ is not distinguished here for clarity. (Inset) schematics of spin structures of three magnetic ground states, presented in a sequence of ascending pressure. The *n*-glide plane constraint between two helical orders in H*a*-I is broken in the H*a*-II phase.

**Figure 2:**
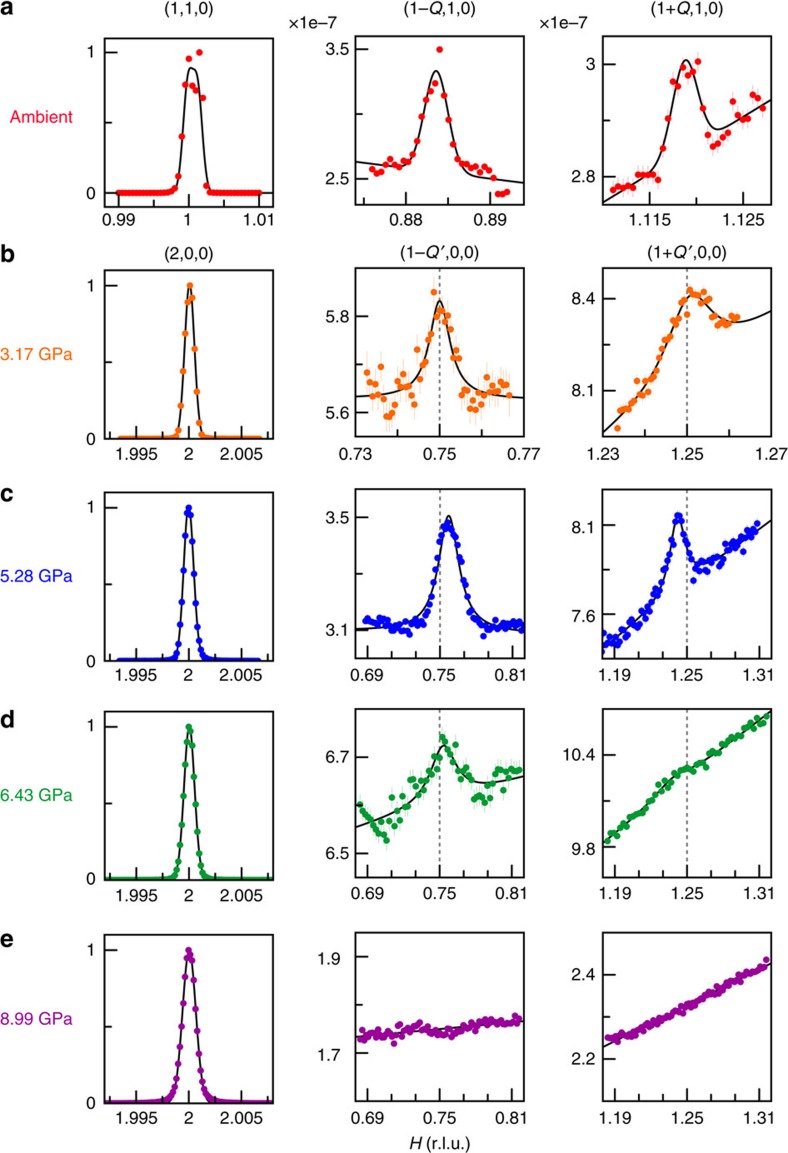
X-ray diffraction evidence of helical order in MnP. (**a**) Raw scans around (1, 1, 0) order at ambient pressure and *T*=4 K, showing both the lattice Bragg peak and a pair of non-resonant magnetic peaks associated with the helical spin order H*a*-I. Solid lines are guides to the eye. (**b**–**d**) Longitudinal (*θ*/2*θ*) line shapes of (2, 0, 0) lattice, and (1±*Q*′, 0, 0) helical magnetic order, measured at *T*=4 K. We set *a*>*b*>*c* in the *Pbnm* space group for the lattice^4^. Lattice line shapes are instrument resolution limited for the whole pressure range, and can be fit to a Pseudo-Voigt form with a lattice coherence length exceeding 1,500 Å. The magnetic peaks are significantly broadened, indicating a shorter correlation length of the helical spin order from ∼310 Å at 3.2 GPa to ∼70 Å at 6.4 GPa, about three times the pitch length of 24 Å. All magnetic peaks are fit with a Lorentzian form on a sloped background, which could be attributed to influence from spin fluctuations in the ordered phase. However, our counting statistics are not sufficient to make a distinction from a Lorentzian-squared form, which results from disorder pinning^45^. The reduced background benefits from the use of a pair of wide-angle perforated diamond anvils^22^,^24^. Vertical dashed lines mark the commensurate (0.75, 0, 0) and (1.25, 0, 0) positions. Our instrument resolution is fine enough to indicate that the observed magnetic pairs are mirror symmetric to the (1, 0, 0) order, but not commensurate. The presence of mirroring peaks around (1, 0, 0) indicates the *n*-glide plane constraint is broken for the spin arrangement at high pressure 4, although the (1, 0, 0) lattice order is still forbidden. (**e**) Above *P*_c_=6.7 GPa, magnetic diffraction is no longer observed in longitudinal scans at same positions of **b**–**d**. Vertical error bars represent 1*σ* s.d. counting statistics.

**Figure 3:**
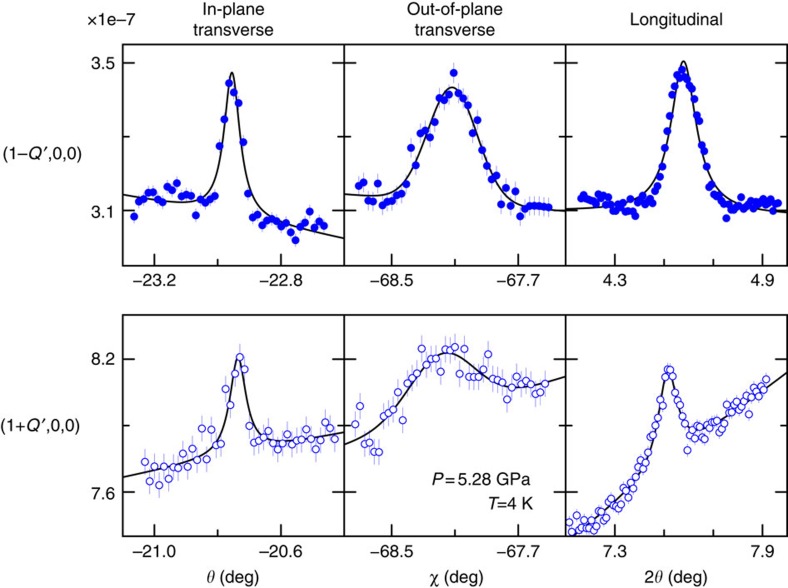
Single crystal nature of the magnetic order at *P*=5.28 GPa. The single crystal nature of the magnetic order is proven by independent raw scans across the 3D reciprocal space for both (1−*Q*′, 0, 0) and (1+*Q*′, 0, 0) orders. The out-of-diffraction-plane transverse scan is dominated by the resolution function determined by the wide horizontal detector slits, while the in-plane transverse scan is intrinsic to the sample mosaic (full-width at half-maximum∼0.1°) under pressure. The longitudinal scans are of the *θ*/*2θ* type (plotted against 2*θ* here) and identical to those in Fig. 2c. Measurements were performed at *T*=4 K. Vertical error bars represent 1*σ* s.d. counting statistics. Solid lines are guides to the eye.

**Figure 4:**
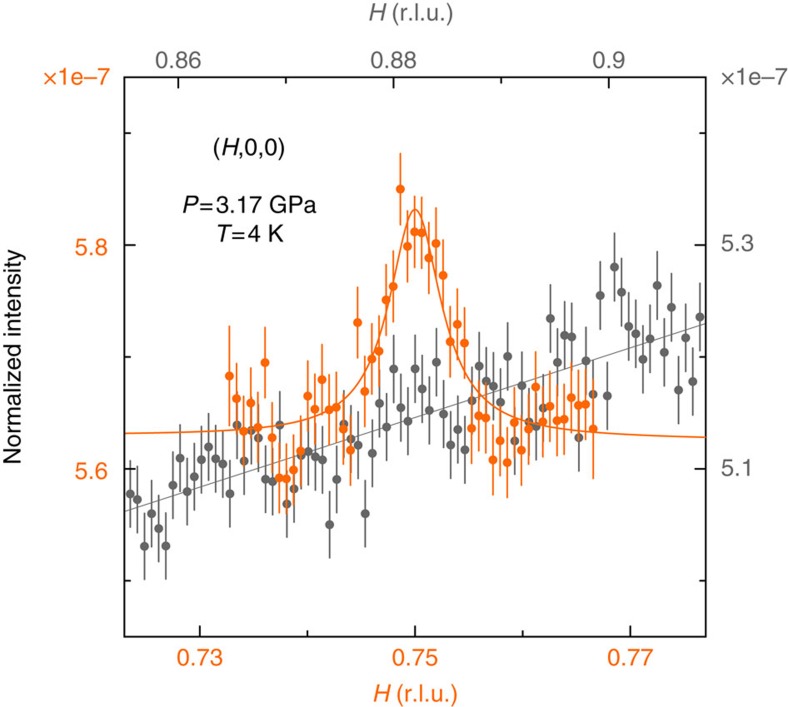
Primary wave nature of the observed diffraction order. A comparison of longitudinal scans between the observed (1−*Q*′, 0, 0) order and null (1−*Q*′/2, 0, 0) position. This indicates that our observed peaks are primary waves and not higher harmonics of another wave vector. The longitudinal scan of (1−*Q*′, 0, 0) is identical to the data in Fig. 2b. Vertical error bars represent 1*σ* s.d. counting statistics.

We performed non-resonant single crystal X-ray magnetic diffraction under pressure (see ‘Methods' section)^20^,^21^,^22^,^23^,^24^ to elucidate the cascade of magnetic states in the *P–T* phase diagram of MnP (Fig. 1) and their relation to superconductivity. We discover helical magnetic order with **Q′**∼(0.25, 0, 0) presaging the high-pressure superconductor (Fig. 2). We observe a pair of superlattice peaks in mirror symmetry to the lattice order at three pressures, 3.17, 5.28 and 6.43 GPa, but absent at *P*=8.99 and 10.4 GPa. These diffraction peaks are always of single crystal nature (Fig. 3) and their pressure evolution is commensurate to that of the *a* axis. Here we adopt the *Pbnm* space group setting for MnP with *a>b>c* (ref. 4). The low transferred momentum of (1−*Q*′, 0, 0) rules out diffraction from integer lattice orders from both MnP and other components of the high-pressure cell (diamond and Ag manometer)^21^,^22^,^23^,^24^. The peak intensities lie in the range of 1–4 × 10^−8^ relative to the (2, 0, 0) lattice intensity, which are comparable with the estimate of non-resonant magnetic diffraction intensities (see ‘Methods' section) and the observed diffraction signal of the low-pressure helical order under the same experimental condition (Fig. 2a). It is known that spin order can induce higher harmonics^25^,^26^. However, we did not observe a diffraction peak at (1−*Q*′/2, 0, 0) with commensurate sensitivity (Fig. 4). This implies that our observed pair of peaks represents the primary wave describing the spin order. We did not observe diffraction intensity at (1−2*Q*′, 0, 0), thereby ruling out a strong charge harmonic to the magnetic order.

Our limited number of observed diffraction orders and the lack of a full azimuthal study because of constrained high-pressure cell geometry make it insufficient to fully refine the high-pressure spin structure. However, in our diffraction geometry, the non-resonant magnetic cross section for orders along the (*H*, 0, 0) direction is only sensitive to magnetic moments projected out of the vertical diffraction plane and transverse to the wave vector **Q**′ (see ‘Methods' section). With spin moments localized in Mn (see below) and an incommensurate wave vector in MnP, the magnetic order is not likely to be of a collinear, amplitude-modulated type. Thus it is reasonable to identify the magnetism in MnP as helical order with tightened pitch (H*a*-II, Fig. 1 inset). This provides a consistent perspective on all three spin structures (H*a*-I, FM and H*a*-II). The spiral magnetism develops with a varying twist angle between neighbouring spin pairs along the wave vector direction, a subtle result due to pressure-dependent, competing exchange constants from multiple close neighbours in an anisotropic lattice^27^. By contrast, a recent nonpolarized neutron diffraction study at *P*=3.8 GPa (ref. 5) suggests spiral order along the shortest axis, *b*, in the *Pnma* space group. This result is surprising since for all other (V/Cr/Mn/Fe/Co/Ni)(P/As/Sb) family members (Table 1, refs 4, 6, 7, 8, 9, 10, 11, 12, 13, 14, 15, 16, 17, 18, 19) the spiral order exists along either of the longer axes, *a* or *c*, in the *Pnma* space group setting.

### Lattice evolution under pressure and magnetostriction

**Figure 5:**
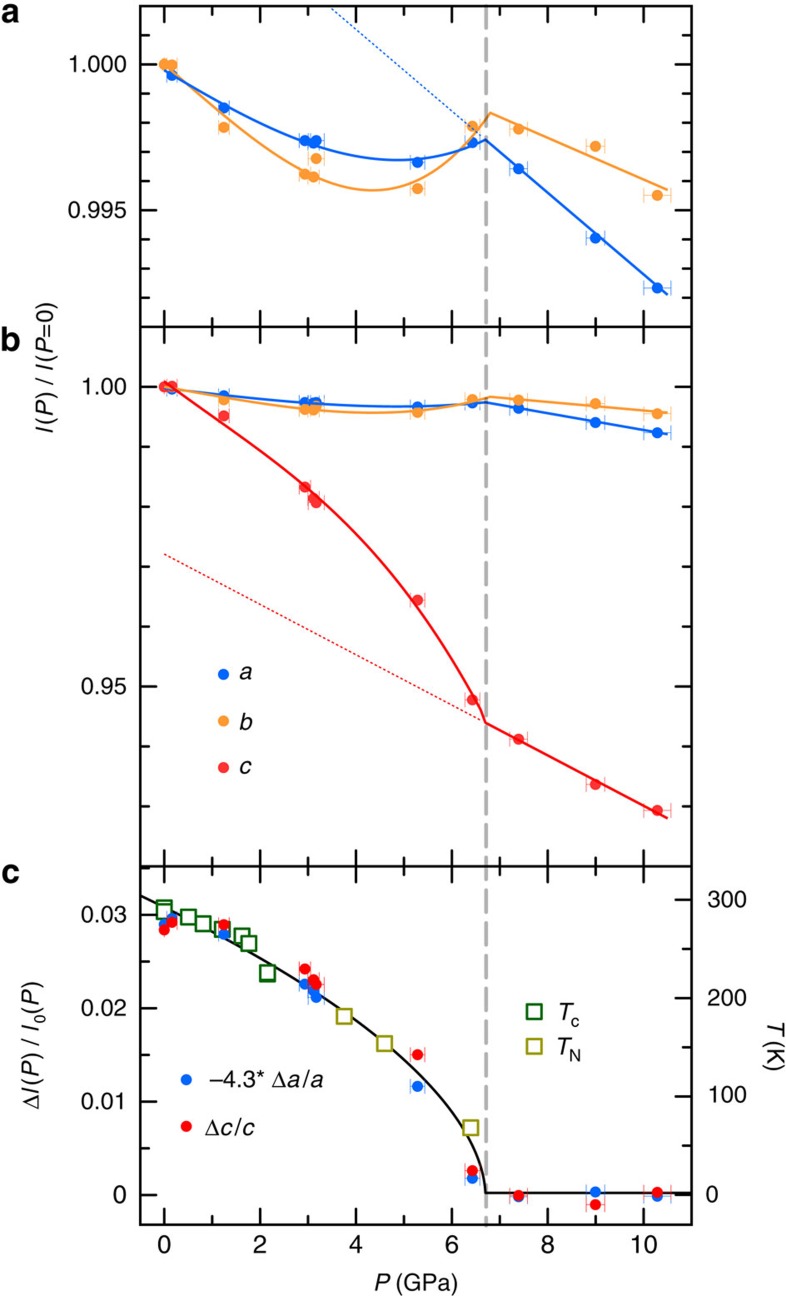
Scaled evolution of the magnetostriction and the magnetic phase boundary in MnP. (**a**,**b**) Normalized lattice evolution at *T*=4 K under pressure, with *a*(*P*=0)=5.8959 Å, *b*(*P*=0)=5.2361 Å and *c*(*P*=0)=3.1807 Å in the *Pbnm* space group. *a*(*P*)/*a*(0) and *b*(*P*)/*b*(0) evolve slowly under pressure and are non-monotonic, while *c*(*P*)/*c*(0) has a strong monotonic pressure dependence. The shapes of *a*, *b* and *c*(*P*) indicate large magnetostriction. Assuming that the lattice of a non-magnetic phase should evolve linearly over this pressure range (dashed lines in **a** and **b** as *a*_0_(*P*)/*a*(*P*=0) and *c*_0_(*P*)/*c*(*P*=0)), and that the low-pressure behaviour can be modelled from extensions of the high-pressure lattice, the magnetostriction is then extracted by subtracting the estimated *a*_0_(*P*) and *c*_0_(*P*). (**c**) Magnetostriction, expressed as Δ*l/l*=(*l*(*P*)−*l*_0_(*P*))/*l*_0_(*P*) in both Δ*c/c* and Δ*a/a*, can be scaled to magnetic phase transition temperatures *T*_C_ and *T*_N_ as a function of pressure. Δ*c* and Δ*a* are of different signs, indicating the anisotropic nature in both magnetic exchange interactions and the lattice's response to the magnetic order. Horizontal error bars represent the full range of pressure during a measurement.

The boundary of the magnetic phase is determined most accurately by the pressure evolution of the lattice. Single crystal refinement of five to six Bragg orders of MnP at each pressure indicates that the lattice structure remains in the orthorhombic phase to 10.4 GPa. Longitudinal scans of lattice orders such as (2, 0, 0), (0, 2, 1), (2, 2, 0) and (2, 2, 2), showing instrument resolution limited profiles with no noticeable peak splitting, support this conclusion. All three lattice constants evolve nonlinearly at low pressure but linearly at high pressure, with the crossover defining the critical pressure, *P*_c_=6.7±0.2 GPa (Fig. 5), consistent with the range where magnetic diffraction was observed directly. The lattice changes continuously under pressure to a sensitivity level of |Δ*l|*/*l*∼1 × 10^−3^ (Fig. 5). The orthorhombic structure of MnP is considered to be a distortion from the hexagonal structure of NiAs (refs 9, 15), as the two symmetries can evolve continuously across the ratio *a*/*c*=1.732. Under pressure, the orthorhombic distortion in MnP, measured by *a*/*c*, keeps increasing from 1.85 to 1.98 and moves away from the hexagonal symmetry. While helical order in both MnSi and CrAs are suppressed by pressure through a clear first-order quantum phase transition^17^,^18^,^28^, the quantum phase transition in MnP at *P*_c_ is isostructural and could be continuous.

The lattice evolution with pressure indicates a significant magnetostriction, which is common to many 3*d* and rare-earth magnetic compounds^29^,^30^. Here in MnP, magnetostriction can be extracted from Δ*c* and Δ*a* of the lattice and scaled to the magnetic phase boundary of either the Curie or Néel temperatures, *T*_C,N_, as Δ*c/c*∼Δ*a/a*∼*T*_C, N_ (Fig. 5c), regardless of whether there is underlying ferromagnetic or antiferromagnetic order. Since the staggered magnetic moment 〈*m*〉 is directly related to the magnetostriction, both Δ*l* and 〈*m*〉 vanish at the quantum phase transition. Beyond *P*_c_, an energy density of 7 GPa distributed over eight valence electrons in the P 3*p* and Mn 3*d* orbitals^31^ increases the electron kinetic energy *t* by ∼15 meV per electron, comparable to the magnetic exchange constants *J* (2.5–11 meV, ref. 32). An increasing *t*/*J* ratio reduces the ordered moment, 〈*m*〉, and eventually destabilizes the magnetism. While 〈*m*〉 drops to zero at a quantum critical point, the fate of individual local moments remains of high interest, as exemplified in heavy fermion materials^33^.

### Local moments and fluctuation modes

**Figure 6:**
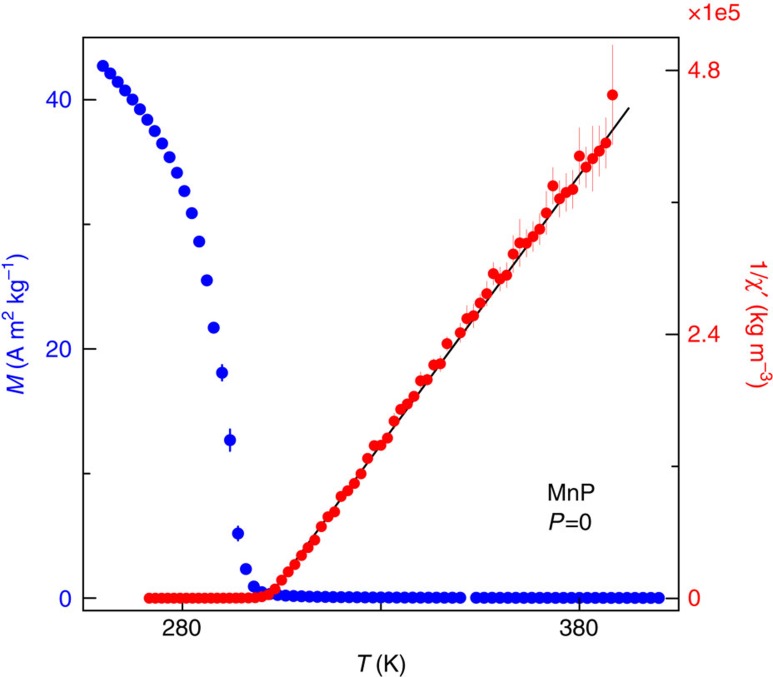
Magnetization and inverse magnetic mass susceptibility at ambient pressure. The magnetization *M* was measured in a SQUID based Magnetic Property Measurement System (Quantum Design) in a 100 Oe d.c. field, and plotted in SI units. Magnetic susceptibility *χ*′(*T*) was fit to the Currie–Weiss law above the ferromagnetic transition at 291 K to extract a moment of 2.79 *μ*_B_ per Mn. The measured Currie–Weiss moment is compared with the literature value of the saturated moment 1.3 *μ*_B_ per Mn (ref. 4) in the high field and low temperature limit to provide a Rhodes–Wohlfarth ratio of 2.2. Vertical error bars represent 1*σ* s.d. of measured magnetization.

Spins in MnP are deep in the local limit at ambient pressure given a Rhodes–Wohlfarth ratio of 2.2 (Fig. 6). The 15 meV per electron increase in kinetic energy sufficient to destabilize the magnetic order is not enough to fully delocalize the 3*d* moments, considering their 0.20 eV bandwidth^31^. Therefore, MnP is a system with local moments surviving beyond the quantum critical point, and spin fluctuations in the disordered state naturally raise special interest about magnetically driven superconductivity.

In the disordered phase, the predominant spin fluctuation modes likely are still dictated by the nearby magnetic instability^33^,^34^,^35^. In MnSi, helical fluctuations in the form of spiral/helix paramagnons were observed for *T*>*T*_C_ despite a weak first order transition. Those fluctuations centre at a wave vector similar in magnitude to the ordering wave vector **Q**, but with a random direction^35^, presumably because of the short range Dzyaloshinskii–Moriya interaction in a cubic lattice symmetry. In MnP and CrAs, the lattice anisotropy likely confines wave vector directions of magnetic fluctuations. The pressure evolution of **Q** in CrAs (ref. 19) is constant up to *P*_c_∼0.65 GPa (refs 17, 18, 19, 36). Interpreting its behaviour for *P>P*_c_ (ref. 36) is clouded by a strong first-order phase transition and a highly strained sample condition (lattice mismatch of several per cent) in the phase coexistence region. With no significant evolution of **Q** in the ordered phase under pressure (Fig. 2)^19^, the disordered phases of MnP and CrAs should possess spin fluctuations dominated by the magnetic instability in the ordered phase, that is, spiral modes centred at **Q**∼(0.25, 0, 0) for MnP and (0.36, 0, 0) for CrAs.

## Discussion

Fluctuation modes in spiral magnets are of particular interest in terms of the competition between spin and lattice (phonon) fluctuations and their connection to superconducting pairing of *s*, *p*, or *d* character. Consider a helical fluctuation at a finite wavelength. By contrast to the usual antiferromagnet, spins of nearest neighbours along the wave vector **Q** direction share a large ferromagnetic projection. These ferromagnetic spin fluctuations in the low frequency limit would suppress phonon-mediated superconductivity due to on-site pairing of itinerant electrons^2^, emphasizing magnetically mediated coupling channels. Furthermore, varying the pitch of the helical order provides a continuous tuning of local ferromagnetic order versus intermediate-range antiferromagnetic order, thus tilting the competition between the two types of magnetically mediated superconductivities.

**Figure 7:**
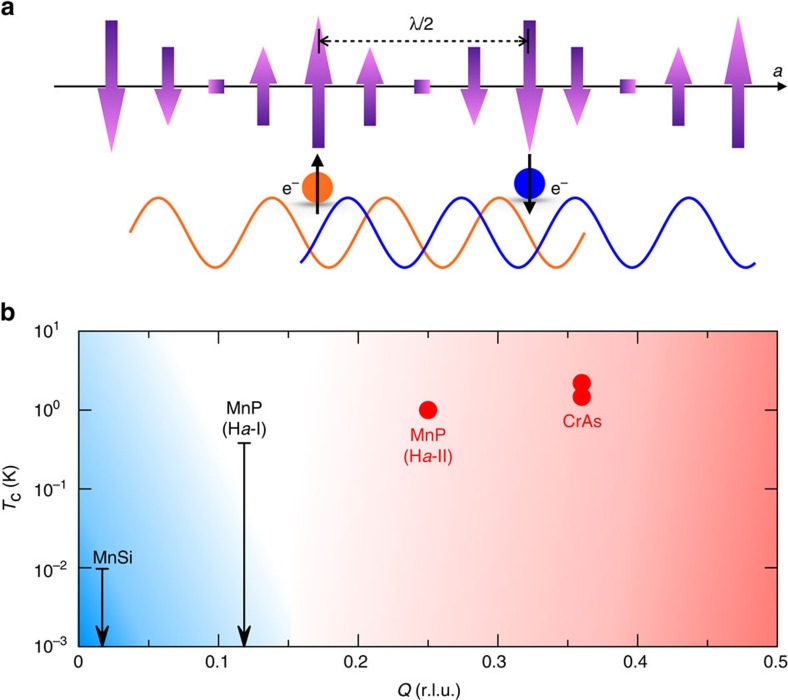
Variable helical pitch length as a tuning method for magnetically mediated superconductivity. (**a**) Schematic of a superconducting electron pair coupled through helical spin order in a projected planar view. The two sites of itinerant electron coupling are separated along the helical order by a half wavelength (*λ*/2), suggesting the possibility of singlet 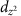-wave pairing. This scenario competes with superconductivity of a ferromagnetic type, while the nearly parallel local spin configuration always suppresses phonon-mediated superconductivity at a single site^2^. (**b**) Superconducting transition temperature *T*_c_ plotted as a function of helical wave vector *Q* in selected 3*d* intermetallic compounds. Data for MnSi (refs 28, 35), MnP (refs 3, 4) and CrAs (refs 17, 18, 19) are collected from either the literature or current work. Red solid circles represent observed superconducting transitions, which only exist in pressure-induced disordered phases beyond the helical order, and are likely antiferromagnetically mediated. The horizontal bars of the downward arrows represent the lower bounds of null searches for superconductivity. Ferromagnetically mediated superconductivity is expected to be at a lower temperature than its antiferromagnetic counterpart^38^,^40^. The pitch of the helical order represents a potential tuning method between ferromagnetically (blue region) and antiferromagnetically (red region) mediated superconductivity.

The spin interaction between two itinerant electrons is an oscillating function in real space, with attractive regions at distance (*n*+1/2)*λ* (where *n* is an integer). The strongest interaction happens at a half pitch length *λ*/2 of the fluctuating spiral modes (Fig. 7a), which is about 12 Å in MnP. This is similar to the antiferromagnetic fluctuation-mediated interaction in the rare earth cuprates and the heavy fermion compounds^37^,^38^. There is a relatively long interaction length between itinerant charge carriers as compared with both the on-site interaction of the phonon-mediated type^37^ and the nearest-neighbor resonant valence bond type for underdoped cuprates^39^. On the other hand, the coherence lengths of Cooper pairs are typically much longer than interaction lengths in both phonon- and magnetically mediated superconductors^37^,^39^ and for MnP, the superconducting coherence length extends over 300 Å (ref. 3). This coherence length is necessarily smaller than the mean free path of itinerant electrons, thereby allowing the electron pair overlap to maintain phase coherence. The MnP samples we used have a residual resistance ratio of ∼1,000 at ambient pressure^3^, close to the clean limit. The issue of pairing symmetry is more tenuous, but the model of helical magnets allows certain predictions. The interaction of paring itinerant electrons at a distance *r*=(*n*+1/2)*λ* along the wave vector **Q** direction of helical spin fluctuations (Fig. 7a) mandates a preferred axial direction and suggests that the superconductivity might be of the singlet 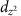 type, especially in light of the low-symmetry lattice structures of MnP and CrAs.

While spiral fluctuations suppress phonon-mediated superconductivity and enhance the coupling channels for the magnetic interactions, helical fluctuations of different pitches provide the means to switch from ferromagnetic to antiferromagnetic character. With increasing spiral wavelength, the interaction strength of the antiferromagnetic coupling is reduced over an elongated *r* (ref. 32). Moreover, an increased spiral wavelength reduces the turning angle between neighbouring spins and thereby heightens the local ferromagnetic spin density. By varying the pitch, it is possible to tune both the ferromagnetic and antiferromagnetic spin fluctuations. Our focus on local moment helical order complements itinerant models of continuous tuning by band filling from ferromagnetic to antiferromagnetic order with a concomitant switch between magnetically mediated superconductivities of different symmetries^40^. Through the comparison of the cuprates and Sr_2_RuO_4_, it appears that ferromagnetically mediated superconductivity typically has an orders of magnitude lower transition temperature than its antiferromagnetic analogue of the same dimensionality^40^.

The dimensionality of the spin fluctuations is another interesting issue. The helical order in 3*d* compounds can be compared with incommensurate antiferromagnetic order in heavy fermion materials like CeCu_6−*x*_Au_*x*_ (ref. 33), where spin fluctuations with two-dimensional character were observed around the ordering wave vector **Q** (ref. 34). Even though the effective low dimensionality enhances the spin fluctuations, the extremely low-magnetic coupling strength in CeCu_6−*x*_Au_*x*_ (ref. 33) suppresses the possible magnetically mediated superconductivity below experimental sensitivity. Spin fluctuations in MnP are likely three-dimensional (3D) judging from the *T*^3/2^ dependence of the resistivity^3^, but they are matched with a large magnetic coupling strength^32^ and bandwidth^31^, so the superconducting transition temperature, *T*_c_, could still be measurable even at a level of *T*_N_/1,000. For 3D helical magnets such as MnP and CrAs with *T*_c_=1–2 K (Fig. 7b), the corresponding ferromagnetic type could be below the lowest range of temperatures measured to date.

Although experimental evidence is still limited, the effects of a variable spiral pitch are suggestive. We illustrate the trends in Fig. 7b for the series MnSi, MnP and CrAs as a function of their different magnetic wave vectors. With a small spiral wave vector of (0.017, 0.017, 0.017)^35^, MnSi does not superconduct under pressure down to at least 10 mK (ref. 28), although the lack of an inversion centre could complicate the symmetry properties of a superconducting state. For MnP at low pressure, the helical order with a wave vector of 0.117 r.l.u. (ref. 4) was replaced by ferromagnetic order at *P*∼1 GPa, and no superconductivity was observed down to 350 mK (ref. 3). On the other hand, both MnP at high pressure (0.25 r.l.u.) and CrAs (0.36 r.l.u.) have relatively large wave vectors (short pitches) and demonstrate superconducting ground states once the helical order is suppressed by pressure^18^,^19^. We list in Table 1 38 different intermetallic compounds with magnetic pitch varying nearly continuously from 0.07 to 0.40 r.l.u. Most of them have not been examined under pressure, neither to map the evolution of their magnetism nor to search for superconductivity. With such studies, the 3*d* helical magnets of the (V/Cr/Mn/Fe/Co/Ni)(P/As/Sb) family^3^,^4^,^6^,^7^,^8^,^9^,^10^,^11^,^12^,^13^,^14^,^15^,^16^,^17^,^18^,^19^ present manifest opportunities to further our understanding of the linkage between magnetism and unconventional superconductivity.

## Methods

### Non-resonant X-ray magnetic diffraction

The technique of non-resonant X-ray magnetic diffraction has been well established at synchrotron-based X-ray sources^29^,^30^,^41^,^42^,^43^. Non-resonant single crystal X-ray magnetic diffraction under pressure using 20.000 keV X-rays was carried out at beamline 4-ID-D of the Advanced Photon Source^20^,^21^,^22^,^23^,^24^. The X-ray energy is calibrated to the K-edge of the element Molybdenum to within a precision of 0.5 eV. The bandwidth of incident X-rays is ∼2.7 eV full-width at half-maximum, given by the use of Si (1,1,1) crystals as X-ray monochromators. Diffraction was carried out in the vertical scattering plane, with X-rays predominantly (>99%) polarized linearly in the horizontal direction perpendicular to the diffraction plane.

MnP is a 3*d* transition metal compound with low local symmetry at the Mn sites. Thus it is reasonable to assume that the orbital moments are quenched in this system. Spins localized at Mn sites, as suggested by our measured Rhodes–Wohlfarth ratio, are the major source of magnetism. Under the non-resonant condition and our vertical diffraction geometry, the cross section of X-ray magnetic diffraction is^41^,


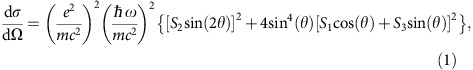


where *e* and *m* are the electron mass and charge, respectively, *ħω* is the X-ray energy, 2*θ* is the Bragg diffraction angle and *S*_1,2,3_ are projections of the reciprocal space spin density with *S*_2_ out of the diffraction plane and *S*_1,3_ in plane^41^. However, the diffraction 2*θ* angles are all small (Fig. 3) given the need to use hard (20 keV) X-rays to penetrate the diamond anvil cell. Expanding the scattering cross section by powers of sin^2^(*θ*)∼0.005, it simplifies to 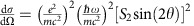, where only the *S*_2_ perpendicular spin projection remains^42^,^43^. In our measurements (Fig. 2), the diffraction wave vectors (1±*Q*′, 0, 0) are parallel to the magnetic order vector (*Q*′, 0, 0). Hence the observed diffraction signals indicate a transverse component of antiferromagnetic order, ruling out a purely longitudinal spin wave. A spiral form is the simplest model that is consistent with our data, since localized spins also rule out an amplitude-modulated, collinear wave form.

The magnetic diffraction cross section, *σ*_mag_, can be compared with the charge diffraction cross section, *σ*_charge_, as roughly: 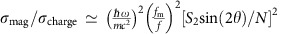, where *N* is the total number of electrons per atom that contribute to the Thompson scattering amplitude, and *f*_m_, and *f* are magnetic and charge form factors, respectively. With *N*=25 for Mn, and a projected spin moment *s*_⊥_ about 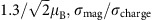 is estimated to be 2–5 × 10^−8^ for *I*_(1±*Q*′, 0, 0)_/*I*_(2, 0, 0)_. This value becomes smaller as the ordered moment, 〈*m*〉, decreases with increasing pressure.

Although the non-resonant X-ray magnetic diffraction signals are typically weak, the feasibility of this technique has been demonstrated under high pressure^20^,^22^,^23^,^24^. With the development of wide-angle perforated diamond anvils^22^,^24^, it is possible to cleanly detect magnetic order with spin moments as low as 0.3 *μ*_B_ under pressure, with the sample volume spatially divided into six magnetic domains^24^. With only 0.9 mm thick diamonds and a 0.1 mm thick low-Z glassy pressure medium (methanol:ethanol 4:1 mixture) in the X-ray scattering path, in addition to the sample, the background was minimized and is devoid of sharp features across reciprocal space.

### Data availability

The data that support the findings of this study are available from the corresponding authors upon request.

## Additional information

**How to cite this article:** Wang, Y. *et al*. Spiral magnetic order and pressure-induced superconductivity in transition metal compounds. *Nat. Commun.* **7,** 13037 doi: 10.1038/ncomms13037 (2016).

## Figures and Tables

**Table 1:** Spiral orders in the (V/Cr/Mn/Fe/Co/Ni)(P/As/Sb) family.

Compounds	*T*_N_(K)	*Q* (r.l.u.)	Helical axis:	Reference
Mn_0.65_Cr_0.35_As	195	0.071	*a*	13
Mn_0.7_V_0.3_As	142	0.08	*a*	8
Mn_0.7_Cr_0.3_As	202	0.088	*a*	13
Mn_0.75_Cr_0.25_As	205	0.097	*a*	13
MnAs_0.925_ P_0.075_	232	0.10	*a*	11
Mn_0.95_Co_0.05_P	53	0.101	*c*	14
Mn_0.9_Co_0.1_P	49	0.107	*c*	14
Mn_0.95_V_0.05_P	107	0.109	*c*	14
Mn_0.8_Co_0.2_P	70	0.111	*c*	14
Mn_0.9_Cr_0.1_P	50	0.112	*c*	14
Mn_0.95_Fe_0.05_P	62	0.113	*c*	14
Mn_0.95_Cr_0.05_P	53	0.116	*c*	14
Mn_0.9_V_0.1_As	206	0.116	*a*	8
MnP (low pressure)	50	0.117	*c*	4
Mn_0.8_Cr_0.2_As	208	0.120	*a*	13
Mn_0.95_V_0.05_As	200	0.128	*a*	8
Mn_0.9_Cr_0.1_As	210	0.133	*a*	13
Mn_0.95_Fe_0.05_As	211	0.142	*a*	10
Mn_0.9_Fe_0.1_P	172	0.145	*c*	14
Mn_0.9_V_0.1_P	152	0.151	*c*	14
Mn_0.95_Ni_0.05_As	202	0.155	*a*	12
Mn_0.95_Co_0.05_As	196	0.166	*a*	9
Mn_0.9_Co_0.1_As	174	0.184	*a*	9
Mn_0.85_V_0.15_P	141	0.189	*c*	14
Mn_0.8_V_0.2_P	113	0.194	*c*	14
FeP	125	0.20	*c*	6
Mn_0.85_Co_0.15_As	152	0.209	*a*	9
Mn_0.8_Fe_0.2_P	142	0.210	*c*	14
MnP (high pressure)		0.250	*c*	Current work
Mn_0.6_Cr_0.4_As	232	0.252	*c*	13
Mn_0.72_Fe_0.28_P	173	0.258	*c*	14
CrAs	265	0.356	*c*	19
Cr_0.98_Ni_0.02_As	202	0.357	*c*	12
FeAs	70	0.395	*c*	16
CrAs_0.86_Sb_0.14_	340	0.40	*c*	7
CrAs_0.72_Sb_0.28_	340	0.40	*c*	7
CrAs_0.66_Sb_0.34_	310	0.40	*c*	7
CrAs_0.5_Sb_0.5_	175	0.40	*c*	7

Organized by antiferromagnetic *Q*-vector from 0.07 to 0.40 r.l.u. in ascending order, all helical orders propagate along either the *a*- or *c*-axes in the *Pnma* space group setting.
